# Elevated Circulating Interleukin 33 Levels in Stable Renal Transplant Recipients at High Risk for Cardiovascular Events

**DOI:** 10.1371/journal.pone.0142141

**Published:** 2015-11-06

**Authors:** Holly Mansell, Mahmoud Soliman, Hamdi Elmoselhi, Ahmed Shoker

**Affiliations:** 1 College of Pharmacy and Nutrition, University of Saskatchewan, Saskatchewan, Canada; 2 St. Paul's Hospital, Saskatchewan Renal Transplant Program, Saskatoon, SK, Canada; 3 Department of Medicine, University of Saskatchewan, University of Saskatchewan, Saskatchewan, Canada; George Washington University School of Medicine and Health Sciences, UNITED STATES

## Abstract

**Background:**

The Major Adverse Cardiovascular Events calculator (CRCRTR-MACE) estimates the burden of cardiovascular risk in renal transplant recipients (RTR). Our recent study of 95 RTR reported the 7-year median risk of cardiovascular events (CVE) to be 9.97%, ranging from 1.93 to 84.27%. Nearly a third (28.4%) of the cohort was above 20% risk for a CVE. Since interleukins (ILs) as part of the inflammatory response may play a role in the pathogenesis of cardiovascular disease (CVD), we extended this study to identify which ILs are associated with high cardiovascular risk in this population.

**Methods:**

Twenty-two ILs were measured by multiplexed fluorescent bead-based immunoassay in 95 RTR and 56 normal controls. Stepwise analysis after multivariate determination of significant demographic and inflammatory variables was performed between the high and low-CVD risk groups (which were arbitrarily set at scores <10% and ≥20%, respectively). Normalized data was presented as mean ± SD and non-normalized data as median (minimum–maximum). Significance was measured at <0.05.

**Results:**

27.5% of the low-risk and 31.3% of the high-risk groups had mean IL levels above the 95 percentile of the normal control levels. In the non-parametric analysis IL-6, 9, 16, 17 and 33 were significantly higher in the high-risk group compared to the control. Univariate analysis (UVA) of the high-risk group identified IL-33 as the only IL that remained significantly higher than the control and low-risk groups (p = 0.000). The percentage of patients with IL-33 levels above the 90 percentile of control value in the low and high-risk groups were 15.6% and 52.0%, respectively (p<0.002). UVA of factors significant to high IL-33 levels included estimated glomerular filtration rate (eGFR), while diabetes mellitus, serum phosphorus, microalbuminuria and age also remained significant in the multivariate analysis.

**Conclusion:**

Circulating IL-33 level is positively associated with high CRCRTR-MACE score. Diminished eGFR, age, diabetes, serum phosphorus and microalbuminurea demonstrate significant relationship with elevated IL-33 levels, supporting the possible pathognomonic role of IL-33 in the cardiovascular burden in RTR.

## Introduction

Risk prediction algorithms are often used in clinical practice to screen for patients at an increased risk of experiencing cardiovascular events (CVE) [1]. In addition to identifying individuals who may require preventative medical intervention, prediction scores have also been used as a surrogate marker of cardiovascular burden in clinical studies. While measuring hard endpoints is obviously the gold standard, enumerating actual CVE requires larger populations and longer observation times. The cardiovascular risk calculator for renal transplant recipients (CRCRTR-MACE) [2,3], is a clinical tool used to estimate the patient’s risk of experiencing a CVE within the next 7 years based on the clinical variables of patient age, low density lipoprotein (LDL), creatinine, presence of diabetes, smoking, previous coronary heart disease and number of transplants. Predicting and managing cardiovascular risk is of particular importance in this population, since cardiovascular disease (CVD) is the leading cause of morbidity and mortality in renal transplant recipients (RTR).

Inflammation plays a pivotal role in vascular injury [4–7]. A significant correlation between circulating pro-inflammatory cytokines and atherosclerosis is well reported in several populations [8,9] including patients on hemodialysis [10] and RTR [11]. This study extends our recent work, where we examined the relationship between thrombopoietin levels and CRCRTR-MACE scores in RTR [12]. Identifying cytokines involved in the pathology of inflammation is an initial step in identifying inflammatory targets to potentially improve the management of CVD. However, since systemic inflammation is inherently present in kidney transplant recipients [11] it is challenging to determine the contribution of each inflammatory mediator to CVD. In this study we set out to identify which inflammatory interleukins were associated with high cardiovascular risk, using the surrogate endpoint of high CRCRTR-MACE.

## Materials and Methods

The protocol was approved by the Regional Ethics Board at the University of Saskatchewan (Bio #11–220). Stable RTR (at least 18 years of age) followed in one out-patient clinic between July 2011 and February 2012 that performed routine blood tests on the same day were eligible for participation. None of the transplant donors were from a vulnerable population and all donors or next of kin provided written informed consent that was freely given. Children were not included in the study. The following subjects were excluded from participation: those requiring hospitalization or a change of more than 10% in serum creatinine in the previous three months, treatment for any acute illness, apparent infection, biopsy proven BK viral nephropathy, as well as those with donor specific antibodies as tested using a magnetic bead-based multiplex assay system, known cancer or pregnancy. Written consent was taken from participants and plasma samples were collected for analysis of inflammatory markers. Patient demographics (age, height, weight, sex, race), cause of kidney failure, mode of dialysis prior to transplant, medications, medical history (diabetes, CVD and events), family history (premature CVD), and smoking history, recent blood pressure, cholesterol, electrolytes (calcium, phosphate, and magnesium), creatinine, urea, albumin, microalbuminuria, parathyroid hormone, vitamin D level, haemoglobin, haemoglobin A1C (HbA1c), left ventricular ejection fraction (LVEF) were obtained from the patient record. The estimated glomerular filtration rate (eGFR) was calculated from the following equation: GFR (CKD-EPI) = 141X min(Scr/k,1)^α^ X max(Scr/k,1)^-1.209^ X.993^Age^ X1.018 [if female] X (1.159 [if black] where k = .7 if female, k = .9 if male, α = -0.329 if female, α = -0.411 if male, min = minimum of Scr/k or 1 and max = maximum Scr/k or 1, Scr = serum creatinine (mg/dL). The 7-year CRCRTR-MACE scores were calculated from published formula [3]. Stratification for CV-risk was defined as low-risk (<10%), moderate risk (10–19%), and high risk (≥20% risk of a CV-event in 7 years).

Blood was collected from a convenience sample to be used as healthy control for the interleukins analyzed in the study. The convenience sample of healthy controls consisted of employees and visitors to our program including family members and friends of transplant recipients. Volunteers were excluded if they were smokers, were under treatment for any acute illness or had infection, cancer or pregnancy, diabetes, or had experienced previous cardiovascular (CV) events.

### Multiplexed fluorescent bead-based Immunoassay

Plasma from 95 RTR and 56 normal controls was frozen at -80 until time of immunoassay (Luminex®) measurement. Two magnetic bead-based multiplex kits {(Bio-Plex Pro Human Cytokine group I panel 27- Plex, Cat # M 50- OKCAF0Y, Bio-Rad Laboratories Canada Ltd, Mississauga, ON, Canada) and (Milliplex map human Cytokine/chemokine panel II 23- plex cat# MPXHCYP2-62K,EMD Millipore, Billerica, MA, USA)} were used to measure the 22 interleukins in this study. The Bio-Plex 200 instrument and Bio-Plex Manager Software (version 6.1) was used for the analysis. Detection limits reported by the manufacturer enabled us to perform the statistical comparisons.

### Statistics

SPSS version 22® (IBM Corp., Armonk, NY, USA) was used for the data collection and analysis. Results were presented as the mean ± standard deviation (SD) for normally distributed data and median (min-max) for non-normally distributed data. All of the hypotheses tested were 2-tailed, and a p value < 0.05 was considered significant. Group differences were analyzed by Student’s t-test and Mann–Whitney U-test for normally and non-normally distributed variables, respectively. Normality was assessed by Shapiro-Wilk test (because the data was less than 2000). Relationship between CRCRTR-MACE scores and other contributing factors (e.g., creatinine, eGFR, etc.) was assessed by Pearson (Spearman) correlation analysis. We used two models of univariate (UVA) and multivariate (MVA) regression analyses to determine the relationship between patient demographics, inflammatory markers, and calculated CV risk according to CRTRTR-MACE. In the first model, CRCRTR-MACE scores were treated as a continuous independent variable. In the second model, CRCRTR-MACE scores were treated as a non-continuous variable at intervals of below or above 20%. Stepwise analyses were performed to determine the contribution of each clinical risk variable. MVA was carried out by stepwise backward elimination by log-likelihood ratio (LR) and conformed by forward LR. The MVA is shown after backward elimination regression of the insignificant variables in both the UVA and an initial multivariate analysis. Finally MVA was carried out with IL-33 as the independent variable to determine what clinical laboratory variables significantly correlates with this interleukin level. Lastly we performed Box plots between IL-33 levels in patients below and above 20% risk for CVE.

## Results

Patient demographics including those at low and high risk for future CVE have been reported recently in detail [12]. In brief, 95 out of 186 participants were deemed stable and participated in the study. The average age was 50.2 years, and 57.4% of RTR were male. The most common reason for exclusion was that the patient was not scheduled for routine blood work on the same day of the clinic appointment, followed by the presence of flu-like symptoms or urinary tract infections. Most patients were on triple immunosuppressive drug therapy, with 83.2% on a mycophenolic acid derivative, 87.4% were on a calcineurin inhibitor, and 85.3% on prednisone. Other immunosuppressants included azathioprine (n = 9), sirolimus (n = 6), leflunomide (n = 3), and belatacept (n = 1). Statin use for hypercholesterolemia was present in 38.9% of patients.

### Cardiovascular risk prediction score

The median 7-year predicted risk according to CRCRTR-MACE was 9.97% (range 1.93–84.2). We categorized high-risk patients, as those predicted to have a ≥20% risk of a CVE, and 28.4% (27/95) of RTR met this criterion according to CRCRTR-MACE.

### Interleukin levels in patients with High and Low CV-risk scores

Results are presented in Table 1. Patients with at least a 20% chance of experiencing a CV event within the next 7 years were classified as high-risk, while patients with less than 10% chance of an event were considered low-risk. The inflammatory profile of each group was compared to each other and controls. Non-parametric analysis between controls and low or high-risk groups identified significant differences in four and seven interleukin levels respectively. Interleukin-33 (IL-33) was the only elevated interleukin in the high-risk group above the low-risk and control groups.

**Table 1:** Comparison between Control, CRCRTR-MACE <10% and CRCRTR-MACE ≥20%.

Marker	Control	MACE < 10%	MACE ≥ 20%	P1	P2	P3	P4
IL6	6.53pg/mL(0.08–58.98)	6.36 pg/mL (0.3–139.31)	9.63 pg/mL (0.3–167.58)	0.140	0.490	**0.036**	0.134
IL8	8.7 pg/mL (0.08–42.28)	11.95 pg/mL (2.85–103.21)	13.52 pg/mL (3.7–495.97)	**0.046**	0.088	**0.049**	0.348
IL9	18.37 pg/mL (0.65–71.8)	23.46 pg/mL (1.72–357.38)	25.11 pg/mL (5.99–165.57)	0.108	0.109	**0.033**	0.328
IL15	2.5 pg/mL (0.59–25.1)	5.2 pg/mL (1.52–20.01)	4.09 pg/mL (1.52–42.79)	**0.012**	**0.003**	**0.010**	0.784
IL16	64.22 pg/mL (5.16–527.73)	88.54 pg/mL (20.57–720.69)	75.28 pg/mL (9.77–392.43)	0.410	**0.018**	0.114	0.571
IL17	31.74 pg/mL (0.09–126.13)	37.46 pg/mL (0.09–167.3)	46.55±27.00 pg/mL	0.250	0.240	**0.042**	0.451
IL20	62.48 pg/mL (18.71–528.16)	195.02 pg/mL (48.8–844.45)	75.05 pg/mL (48.8–806.74)	**0.001**	**0.001**	**0.032**	0.419
IL21	17.37 pg/mL (3.27–30.92)	19.53 pg/mL (3.8–39.74)	19.53 pg/mL (3.21–30.18)	**0.004**	**0.002**	**0.031**	0.809
IL33	19.53 pg/mL (0.14–150.91)	19.53 pg/mL (0.14–53.75)	32.37 pg/mL (0.14–337.65)	**0.000**	0.108	**0.002**	**0.005**
IFN-γ	71.54 pg/mL (0.07–2886.64)	67.5 pg/mL (0.07–1818.3)	102.62 pg/mL (0.07–1432.36)	**0.003**	0.288	0.637	0.279

P1 is the p value between the three groups: control, CRCRTR-MACE <10% and CRCRTR-MACE ≥20%, (P1 was computed by one-way ANOVA); P2 is the p value between control and CRCRTR-MACE <10%; P3 is the p value between control and CRCRTR-MACE ≥20%; P4 is the p value between CRCRTR-MACE <10% and CRCRTR-MACE ≥20% (P2,P3&P4 were computed by Mann-Whitney U-test).

Note the following interleukins were not significant in the analysis: IL1a, IL1b, IL2, IL4, IL5, IL7, IL10, IL12p70, IL13, IL23, IL23A, TNF-α (full results shown in appendix 1)

**Fig 1:**
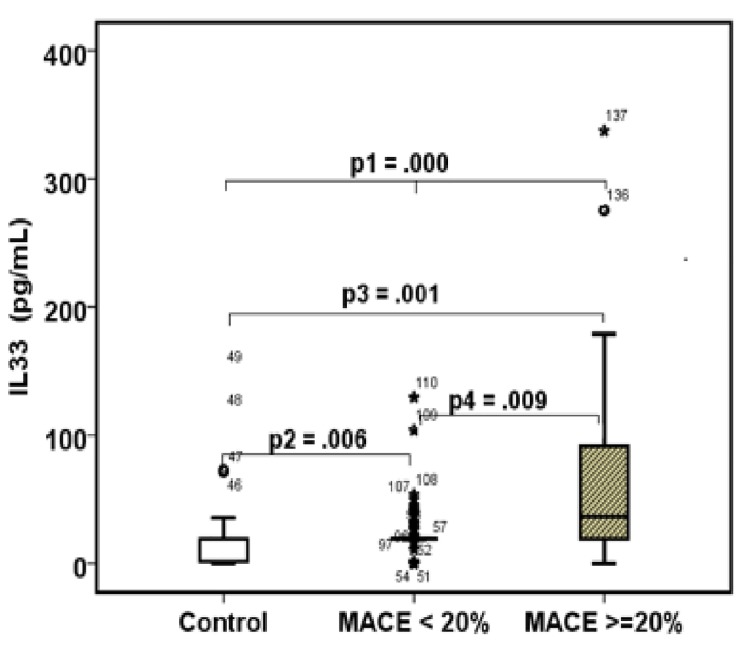
The dark line in the middle of the boxes is the median of IL-33. Half of the cases have a value greater than the median, and half have a value lower. The bottom of the box indicates the 25th percentile while the top represents the 75th percentile. The points are **outliers,** while asterisks or stars indicate **extreme outliers.** p1 (the p value between the three groups) was computed by one-way ANOVA, while p2, p3, and p4 were computed by Mann-Whitney U-test.

Box plots analysis as seen in Fig 1 illustrate the significant elevation of IL-33 in the high-risk compared to patients with a <20% risk, and control group.

### Association between CRCRTR-MACE and Interleukin Levels and Clinical Variables

UVA analysis was performed between the low and high CRCRTR-MACE scores and interleukin levels. Because IL-33 was the only interleukin level significantly associated with high CRCRTR-MACE score was (p = 0.00) (Table 2), no further multivariate analysis was performed.

**Table 2:** Univariate analysis for CRCRTR-MACE scores (low = 0 and High = 1) Vs Interleukins.

Parameter	B	Std. Error	T	P Value	95% Confidence Interval	
					Lower Bound	Upper Bound
Intercept	0.175	0.056	3.113	0.003	0.063	0.286
IL33	0.003	0.001	3.697	0.000	0.001	0.005

The following inleukins were not significantly associated with CRCRTR-MACE in the univariate analysis: IL1a, IL1b, IL2, IL4, IL5, IL6, IL7, IL8, IL9, IL10, IL12p70, IL13, IL15, IL16, IL17, IL20, IL21, IL23, IL28A, IFN-γ and TNF-α

Multivariate analysis was not necessary, since only one interleukin (IL-33) was significant in the univariate analysis.

### Association between IL-33 and Patient Demographics and Laboratory findings

UVA and MVA were then performed to determine which clinical variables significantly associate with IL-33 (Table 3). Patient age, weight, BMI, BSA, diabetes, GFR, SBP, phosphate and urine microalbumin were significantly associated with IL-33 levels in the UVA. Patient age, presence of diabetes and urine microalbumin, serum phosphate remained significant in the MVA, but lost its significance in the stepwise model.

**Table 3:** Univariate, multivariate and stepwise model for IL-33 VS patient demographics.

Parameter	UVA	P Value	MVA	P Value	Stepwise Model	P Value	95% C.I		Estimate SD
							Lower	Upper	
Intercept			417.351	0.217	-73.191	0.010	-128.046	-18.336	
Patient age	1.367	0.001	1.344	0.003	1.191	0.005	0.361	2.021	16.539
Weight	0.825	0.018	10.304	0.102					
BMI	2.958	0.016	-10.781	0.100					
BSA	51.200	0.038	-511.950	0.136					
Diabetes	40.822	0.001	28.282	0.030	25.489	0.037	1.602	49.376	
eGFR(CKD-EPI)	-0.517	0.042	0.143	0.574					
Systolic BP	0.755	0.048	-0.413	0.298					
Phosphate	48.530	0.019	41.218	0.045	32.786	0.085	-4.610	70.182	
Urine microalbumin	0.118	0.005	0.132	0.003	0.117	0.004	0.038	0.196	15.426

The following parameters were not significantly associated with IL-33 levels in the univariate analysis: height, transplant duration, total cholesterol, TC:HDL, TGL, LDL, HDL, hs-CRP, calcium, vitamin D, parathyroid hormone, HbA1c if diabetic, creatinine, urea, Hgb, ejection fraction, smoking, diastolic BP, magnesium, and albumin.

## Discussion

In this study we sought to identify interleukins associated with increased CV risk using a relatively new prediction model [3] developed for RTR. As a side comparator we used a control of healthy normal subjects. Some interleukins were elevated in both patient subgroups (high and low-risk) above control values. They likely contribute to the known systemic inflammatory burden, which is increased in transplantation [13]. In this study we noticed elevated levels of IL-6, IL-8, IL-9, IL-17 and IL-33 in the high risk group.

IL-33 is particularly interesting because it is the only elevated interleukin in the high-risk group above low-risk patients. The implications of IL-33 in inflammation and disease pathology, and role in solid organ transplantation have been previously discussed [14,15,16]. In brief, IL-33, a member of the IL-1 cytokine super family, is up-regulated following pro-inflammatory stimulation. It augments the Th type 2 immune response, and the highest concentrations in humans are observed in fibroblastic reticular, epithelial and endothelial cells. An ambiguous role has been noted for this interleukin, with some studies indicating that IL-33 is protective [17, 18, 19, 20, 21, 22], while other reports suggest a variable protective or pathogenic role depending on the model or the disease process [23]. Other researchers have postulated that it is pathogenic [24, 25,26]. For instance, IL-33 prolonged cardiac allograft survival in murine models, presumably through induction of a Th type 2 immune response [27,28]. It seemed to have a protective role in atherosclerosis in mice [29,30], and was inversely correlated with BMI, exhibiting a protective lipid/metabolic profile in non-diabetic, but not diabetic subjects [31]. Keeping in line with the potential role of IL-33 as a mediator in vascular injury, however, IL-33 levels were significantly increased in the urine of human kidney transplants as soon as 30 minutes after reperfusion (n = 26), and both serum and plasma levels were positively correlated with cold ischemic time, from 30 min to 3 days post-transplant [18]. Further, IL-33 has been shown to promote angiogenesis and vascular leakage [32], with increased levels noted in patients with acute cerebral infarction compared to controls [33]. It has also been implicated in the pathology of acute and chronic kidney disease [34, 35].

The present study favors the notion that IL-33 is likely a marker of injury rather than exerting a protective effect. Several main findings support its potential pathognomonic role in vascular injury. First, IL-33 levels were significantly correlated with CV-risk in RTR. Secondly, IL-33 levels were positively associated with age and microalbuminuria (p = 0.003), two well-known risk factors for vascular injury and cardiovascular events [36,37]. Thirdly, high IL-33 levels were associated with diabetes (p = 0.030) and high serum phosphate levels (0 = 0.045), which are both associated with vascular diseases [38,39]. In fact, serum phosphate levels have been shown to directly correlate with atherosclerosis in both humans [40] and animal models [41], and increased phosphate levels are associated with increased cardiovascular disease and mortality [42]. It is also noteworthy that IL-33 levels were significantly associated with worsening in eGFR in the univariate analysis, although this relationship did not remain strong in the multivariate analysis. Diminished GFR is a well-accepted major risk factor for CVD [43, 44]. Finally, we have also recently measured the levels of soluble ST2 (a decoy receptor which binds to IL-33 and inhibiting cytokine signaling) in this same population. Significantly higher levels of ST2 were found in the high CV-risk group compared to the low risk group (data available on request).

While increased levels of ST2 are recognized as a marker of poor prognosis in patients with myocardial infarction and heart failure, the prognostic value of circulating IL33 levels in CVD has been less clear [45–47]. In a study of 59 patients with acute myocardial infarction no significant relationship was noted between IL-33 and patient prognosis, and levels were similar to the control group [48]. Liu et al investigated serum levels of IL33 and matrix metalloproteinase-28 (MMP-28) (n = 103) and found that serum levels of IL-33 were significantly lower (P< 0.01) and serum concentrations of MMP-28 were higher (P < 0.05) in acute myocardial infarction and unstable angina pectoris compared with stable angina and control groups [49]. Other recent studies have shown that elevated levels of both sST2 and IL33 were associated with increased mortality in ST elevation myocardial infarction (STEMI), but IL33 did not predict mortality in patients with non-ST elevation myocardial infarction (NSTEMI), or stable angina [45,50]. Notably these recent studies used specific enzyme-linked immunosorbent assays (ELISA) to measure circulating Il33, and levels were undetectable in over half of the cohort. By contrast we used multiplexed fluorescent bead-based immunoassay to measure interleukin levels, which confers increased precision and detectability.

In addition to IL-33, this study identified four cytokines (IL-6, IL-8, IL-9 and IL-17) that were significantly increased in the high-risk group, which suggests their potential role in CV pathology. IL-6 has been well studied particularly in the context of atherosclerosis [51]. In the general population, IL-6 may predict mortality in patients with unstable coronary artery disease, and identify candidates that benefit from an early invasive treatment [52,53]. In the setting of myocardial infarction studies indicate that short-term IL6 signaling can protect myocardial tissue in response to acute damage, whereas over the long term increased levels of IL6 play a causative role in CVD [54]. Detecting acute rejection may be another potential role for this interleukin in RTR. In a pilot study (n = 64 total; 32 training sample, 32 validation sample) measuring IL-6 levels allowed for the exclusion of acute rejection with sensitivity and specificity of 92% and 63%, respectively [55]. The atherogenic potential of IL-8 is also well documented. It is suggested that IL-8 down regulates matrix-degrading metalloprotienases-1 expression, which creates an imbalance between MMP and TIMP activities [56]. In a study of 93 RTR, IL-8, (along with age, CRP, and pregnancy-associated plasma protein) was a marker of carotid atherosclerotic plaque measured by carotid ultrasound [57].

In the general population, the role of IL-9 and its receptor have been studied by Gregerson and colleagues [58]. IL-9 plasma levels were significantly raised in patients with carotid atherosclerosis compared with healthy controls (n = 28), and in patients admitted for acute ST-elevation myocardial infarction (STEMI). mRNA levels of IL-9 and IL-9R were also increased in carotid plaques (n = 68) compared to non-atherosclerotic vessels (n = 10), suggesting a role for this interleukin both within the lesion as well as systemically. The role of IL-9 in RTR is currently unknown.

IL-17 has shown to be associated with pro-inflammatory conditions, such as autoimmune diseases and atherosclerosis [59]. It has also been found in the plaques of atherosclerotic lesions [53]. In liver, kidney and lung transplants, IL-17 levels have been associated with an increased risk of graft rejection [60, 61].

Not surprisingly, this study identified some noteworthy differences between the inflammatory profile of RTR and general populations. For example, IL-1 was not elevated in our population as found in general populations [62] with increased CV-risk. Also, it is generally accepted that a trend towards the Th type 1 response, accompanied higher levels of ‘classical’ inflammatory biomarkers is linked to the pathological process of atherosclerosis [63], whereas polarization towards a Th type 2 response tends to predominate in immunologic pathology [64]. The trend towards the either the Th type 1 or 2 response was not observed in RTR.

Several limitations should be mentioned. Our normal subjects provided us with plasma samples that served as a comparison for interleukin levels, but we were unable to ascertain their CV risk score by clinical examination at time of sample collection. We asked all controls about their health, however, and excluded patients with known risk factors for CVD. Because we used the CRCRTR-MACE equation which is used exclusively in RTR, it was not possible for us to match the control group to the RTR according to CV-risk. We therefore opted to use a group of healthy controls with minimal CV-risk as a comparison.

In this study we examined the association between CRCRTR-MACE scores inflammatory interleukins. Our findings suggest (but do not prove) their contribution to CVD, since we used a surrogate marker rather than actual CV events. A prospective study is needed to investigate if these interleukins will have the ability to predict actual cardiovascular events. Currently, the CRCRTR-MACE model is not widely used in transplant recipients. The equation was developed from a population size of 1329 and internally validated on a sample of 701. The authors suggested that care should be taken when applying this model on patients with risk extremes, since high-risk subjects may have been excluded from the ALERT study [3]. Nevertheless the model was externally validated on data from the PORT population [65] (n = 4,146). The model performed reasonably well in the external validation, but CV risk was underestimated in deciles 5 and 9 [4]. We opted do an exploratory analysis with inflammatory markers in association with this particular risk assessment tool, since currently it seems the most promising in this population.

To conclude, the goal of this study was to identify circulating inflammatory interleukins that significantly correlate with high CRCRTR-MACE scores. IL-33 emerged as the only interleukin to positively associate with CRCRTR-MACE score supporting its potential pathognomonic role in the cardiovascular burden in RTR Prospective studies should be undertaken to determine its utility as a biomarker to predict actual CV events, and to further delineate its specific role in this particular population.

## Abbreviations

CRCRTR-MACE: Cardiovascular Risk Calculator for Renal Transplant Recipients (Major adverse cardiovascular events
CV: cardiovascular
CVD: cardiovascular disease
eGFR: estimated glomerular filtration rate
IL: interleukin
LR: log-likelihood ratio
LVEF: left ventricular ejection fraction
MVA: multivariate analysis
OR: odds ratio
RTR: renal transplant recipients
SD: standard deviation
UVA: univariate analysis
